# Severity of sepsis in patients with acute purulent destructive pulmonary disease depending on the presence of type 2 diabetes: impact on the forecast

**DOI:** 10.1186/cc14010

**Published:** 2014-12-03

**Authors:** A Babobekov, B Babadjanov, S Atakov, E Shalaeva

**Affiliations:** 1 Republican Center of Purulent Surgery and Complications of Diabetes, Tashkent Medical Academy, Tashkent, Uzbekistan

## Introduction

Lung abscesses and gangrene are the most severe clinical manifestation and outcome among acute purulent destructive pulmonary disease (APDPD). Mortality ranges from 10 to 35%, and in the presence of diabetes increases up to 30 to 90% [1]. The main reason for this is the generalization of infection (sepsis), leading to the development of multiple organ failure [2,3]. The aim of this study was to identify the severity of sepsis in patients with APDPD depending on the presence of type 2 diabetes, and the impact on the forecast.

## Methods

During the period 2012 to 2013, we examined 408 patients aged 48.5 ± 12.5 years (258 men/150 women) who underwent surgical treatment for APDPD. The patients were divided into two groups: 144 patients with type 2 diabetes, and controls (*n *= 246). We carried out computed tomography, ECG, echocardiography, laboratory biochemical testing, and bacteriological analysis of pathologic material and blood samples.

## Results

**Figure 1:**
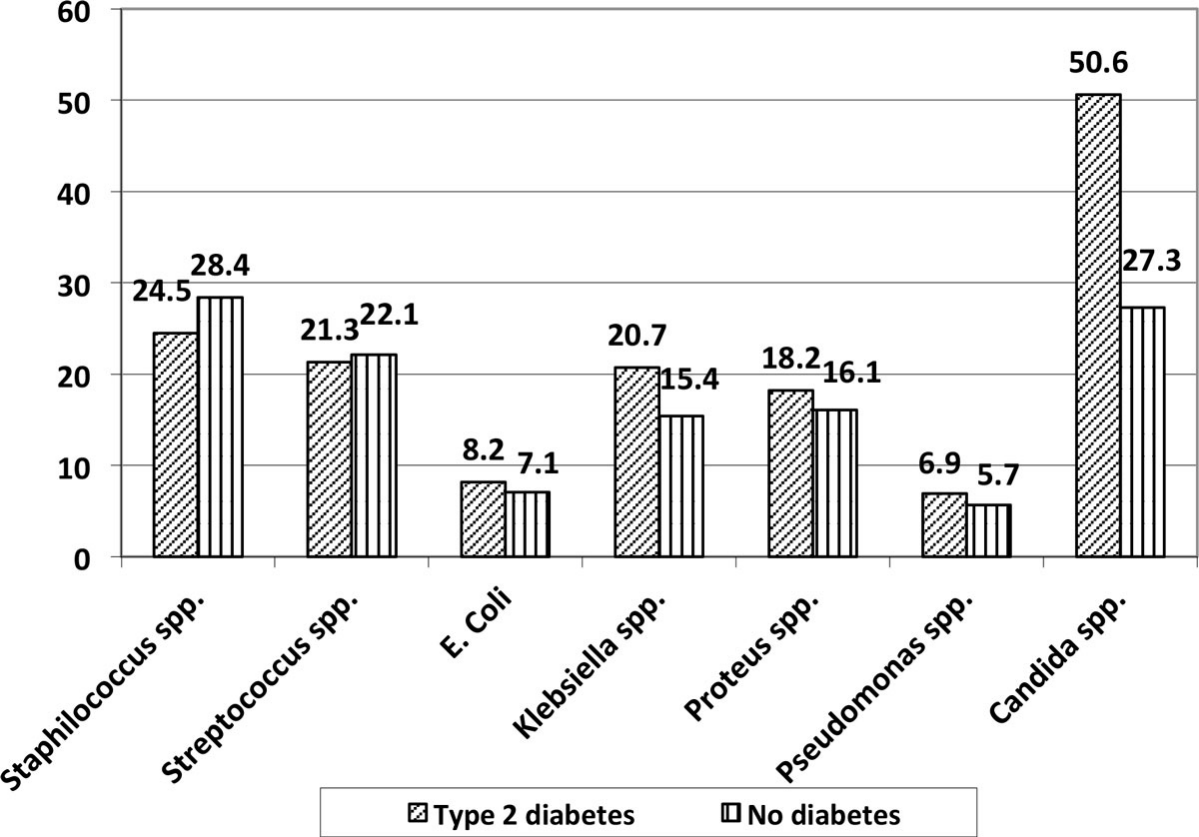


**Figure 2:**
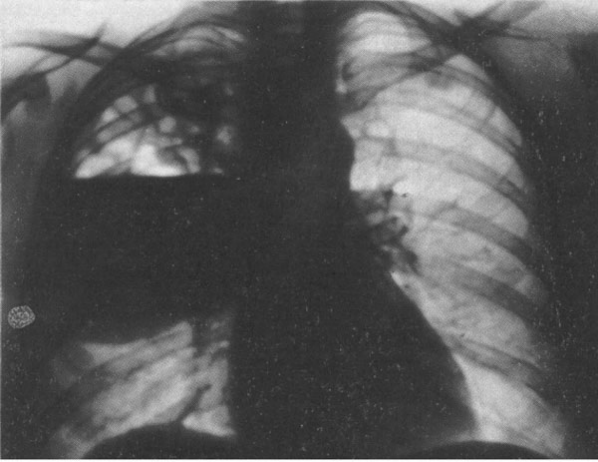
**Man, 42 years old, with right lung abscess after admission to the centre**.

**Figure 3:**
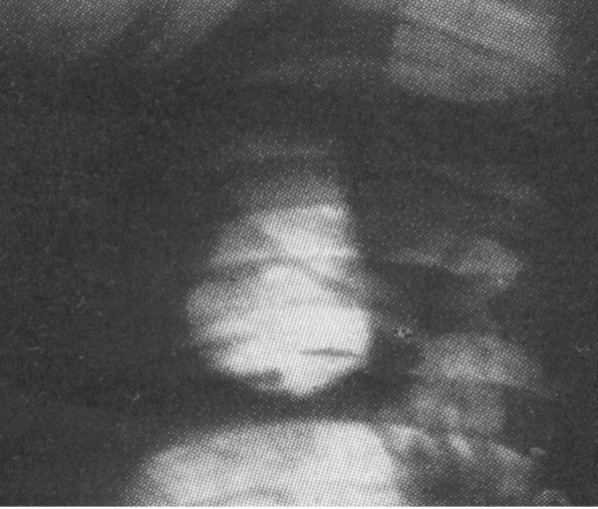
**The same patient on the fourth day after drainage of the abscess**.

**Figure 4:**
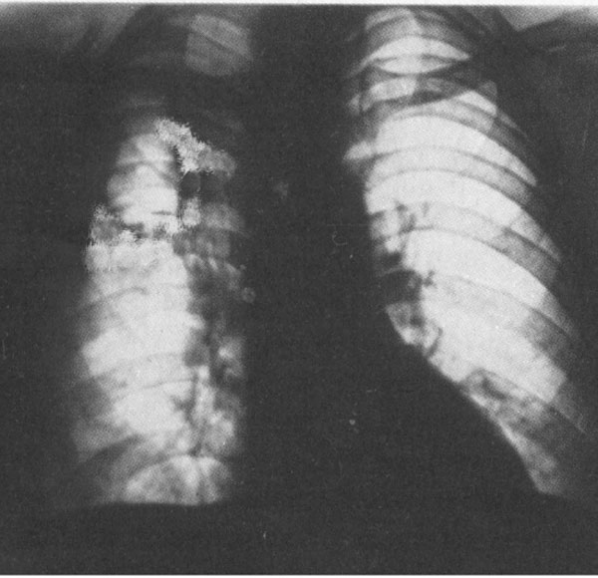
**The same patient before discharge, 14 days in dynamics**.

Patients with type 2 diabetes had much more complications and cases of severe sepsis and septic shock (Table 1). Bacteriological analysis of the pathologic material showed Gram-positive bacteria in 35 to 45%, anaerobic association in 55 to 65%, pathological fungi in 50 to 60%. The patients with type 2 diabetes had much more time from the onset of the first symptoms of lung disease prior to admission (12.5 ± 3.5 vs. 7.5 ± 2.5 days, *P *= 0.002), and the duration of inpatient treatment was significantly longer (13.8 ± 5.5 vs. 7.1 ± 3.4 days, *P *= 0.001). Only 53 (36.8%) of patients with type 2 diabetes and 68 (29.5%) without it had bacteriological positive blood culture. The analysis of the distribution of pathogens in groups is presented in Figure 1. Patients with diabetes had more *Candida *spp. (Figure 1). Figures 2, Figure 3 and Figure 4 present the X-ray dynamics of a 42-year-old man with lung abscess. Clinical recovery in patients with type 2 diabetes was significantly worthy compared to controls (45 (31.2%) vs. 153 (57.9%)), mortality rate 48 (33.3%) versus 39 (14.7%), respectively.

**Table 1:** Clinical symptoms and severity of sepsis in patients with acute purulent destructive pulmonary disease depending on the presence of type 2 diabetes

Data	Type 2 diabetes (*n *= 144)	Control (*n *= 264)
Acute lung abscess	59 (40.9)	122 (46.2)
Necrotizing pneumonia	47 (32.6)	98 (37.1)
Lung gangrene	38 (26.4)	44 (16.7)
Empyema	88 (61.1)	81 (30.7)
Pyopneumothorax	16 (11.1)	9 (3.4)
Mediastinitis	34 (23.6)	16 (6.1)
Body temperature >38°C/<36^°^C	98 (68.1)/21 (14.6)	261 (98.9)/3 (1.1)
Respiratory rate >20/minute	144 (100)	264 (100)
Heart rate >90 beats/minute	138 (95.8)	242 (91.7)
PaCO_2 _<32 mmHg	144 (100)	264 (100)
Leukocytes >12,000/<4,000 cells/mm^3^	111 (77.1)/13 (9.1)	202 (76.5)/11 (4.2)
Renal failure, oliguria	42 (29.2)	34 (12.9)
Increase liver enzymes	34 (23.6)	45 (17.1)
Systolic blood pressure <90 mmHg	33 (22.9)	51 (19.3)
Sepsis	101 (70.1)	223 (84.5)
Severe sepsis	25 (17.4)	29 (11.0)
Septic shock	18 (12.5)	12 (4.5)

Data presented as *n *(%).

## Conclusion

In patients with acute purulent destructive pulmonary disease and type 2 diabetes, severe sepsis and septic shock more often prevailed, inpatient mortality rate was 2.27 times higher, compared to patients with normal glucose metabolism.
